# Supplementary material for: Risk assessment of arteriovenous fistulas focusing on the relationships between the properties of shunted blood flow sounds and a medical/surgical history of hemodialysis patients
Source: PLoS One. 2025 May 23;20(5):e0324747. doi: 10.1371/journal.pone.0324747 (PMC12101638; doi:10.1371/journal.pone.0324747)
Supplement: Attachment — Submitted filename: Response to Reviewers.pdf [file pone.0324747.s008.pdf]

Response to journal requirements

No.	Journal requirements	Responses
1	Please ensure that your manuscript meets PLOS ONE's style requirements, including those for file naming.	Thank you for your guidance regarding PLOS ONE's style requirements. We have carefully reviewed and revised our manuscript to ensure it aligns with the journal's guidelines, including those pertaining to file naming conventions. All revisions have been highlighted in blue for your convenience. If there are any additional areas that require further adjustments, we respectfully request your feedback.<br>•We started a new line numbering sequence from the Abstract onward.<br>•Annotations for figures and tables have been marked with symbols, and new captions have been added.
2	Please state what role the funders took in the study. If the funders had no role, please state: "The funders had no role in study design, data collection and analysis, decision to publish, or preparation of the manuscript."	We have included the following statement in the cover letter to address this requirement:<br>"The funders had no role in study design, data collection and analysis, decision to publish, or preparation of the manuscript."
3	Please include captions for your Supporting Information files at the end of your manuscript, and update any in-text citations to match accordingly.	After careful review, we confirm that our manuscript does not include any Supporting Information files, as all necessary data, figures, and tables are presented within the main text. No additional captions or citations are required. We have reviewed the manuscript to ensure clarity and to avoid any potential confusion regarding supplementary materials.

Response to reviewer comments

We appreciate the reviewer's insightful comments and valuable suggestions, which have significantly improved the quality of our manuscript. Below, we provide a detailed response to each comment.

No.	Reviewer comments	Responses and revisions
1	I suggest to include the following paper:https://doi.org/10.3390/life14111382.	We have incorporated the content of the suggested paper in lines 39-41 of the manuscript. Further, to align with this inclusion, we have added a related statement to lines 44-45. The added content is highlighted in red in the "Revised Manuscript with Track Changes" file. For your reference, the added content is as follows (underlined below)<br>•When AVF failure occurs, percutaneous transluminal angioplasty, thrombus aspiration, or vascular stenting procedures are performed. Ongoing research aims to develop safer and more efficient methods for these interventions [6-8]. (Lines 39-41)<br>•Therefore, it is essential to enhance self-management skills and enable the early detection of abnormalities before the need for endovascular treatment or AVF reconstruction arises. (Lines 44-45)
2	The language must be reviewed in details.	The language throughout the manuscript has been thoroughly reviewed and revised to improve clarity and readability. Since the revisions cover the entire manuscript, all changes have been highlighted in green in the "Revised Manuscript with Track Changes" file. We hope the revised manuscript addresses your concerns effectively.
3	The abstract is not clear, I suggest to improve the structure.	We have completely revised the abstract to improve its clarity, structure, and alignment with the study's objectives and findings. The revised version provides a clear and concise summary of the study's background, methods, results, and conclusions. We hope that the updated abstract addresses your concerns effectively.
4	Methods are clear but the language must be improved.	As noted in Comment 2, the "Methods" section has undergone a thorough language review and revisions to enhance its readability. Since these revisions extend throughout the entire "Methods" section, all changes have been highlighted in green in the "Revised Manuscript with Track Changes" file for your reference.
5	Discussion must be reviewed with a comparation between your results and similar research.	To address this comment, we have incorporated comparisons with relevant studies in the Discussion section. While we searched for prior studies directly associating AVF sounds with physical characteristics, we could not identify any. Therefore, we referred to

		literature demonstrating associations between diabetes and AVF patency rates and made the following adjustments to Lines 330-338 ;<br>"According to previous studies, diabetes mellitus has been shown to contribute to arteriosclerosis [23], and AVFs in diabetic patients have lower patency rates than in those without diabetes mellitus [24,25]. Arteriosclerosis is considered a precursor to vascular stenosis [26], and the frequency of hemodialysis is associated with an increased risk of AVF failure [27,28]. The findings of the present, combined with prior research, it is suggested that diabetes mellitus-induced arteriosclerosis and a prolonged AVF vintage of five years or more negatively affect the vascular intima, resulting in higher TMP values. Therefore, combining TMP data with information on diabetes mellitus history and AVF vintage may provide valuable insights into the presence of AVF thickening or stenosis."<br>These additions aim to provide a broader context for the study findings and align them with existing research.
