# Supplementary material for: Risk assessment of arteriovenous fistulas focusing on the relationships between the properties of shunted blood flow sounds and a medical/surgical history of hemodialysis patients
Source: PLoS One. 2025 May 23;20(5):e0324747. doi: 10.1371/journal.pone.0324747 (PMC12101638; doi:10.1371/journal.pone.0324747)
Supplement: Attachment — Submitted filename: Response_to_Reviewers_auresp_3.pdf [file pone.0324747.s010.pdf]

# Response to journal requirements

| No. | Journal requirements | Responses |
|---|---|---|
| 1 | Please review your reference list to ensure that it is complete and correct. If you have cited papers that have been retracted, please include the rationale for doing so in the manuscript text, or remove these references and replace them with relevant current references. Any changes to the reference list should be mentioned in the rebuttal letter that accompanies your revised manuscript. If you need to cite a retracted article, indicate the article's retracted status in the References list and also include a citation and full reference for the retraction notice. | Thank you very much for your valuable comments regarding the references in our manuscript. We have thoroughly reviewed the reference list to ensure its completeness and accuracy. Please note that no retracted articles have been cited. All revisions have been clearly highlighted in blue. Specifically, we have made the following changes:<br>• The DOI has been added to Reference No. 21 to ensure completeness and accuracy.<br>• To ensure accurate citation, "et al." has been removed from the author list in Reference No. 21.<br>• We have corrected the volume number of Reference No. 30 from 43 to 53.<br>We appreciate your attention to detail and hope that these revisions now meet the journal's requirements. |

# Response to editor and reviewer comment

| No. | Editor and reviewer comments | Responses and revisions |
|---|---|---|
| 1 | • Take professional help to revise / edit the language.<br>• The paper is interesting but I suggest to revise the language. | Thank you very much for your valuable comments regarding the language. We have had the manuscript professionally revised by experts in both English and our native language. Below are the main revisions.<br>• We have revised the title of our manuscript from "Risk assessment of arteriovenous fistulas using arteriovenous fistula blood flow sounds and physical characteristics of hemodialysis patients" to "Risk assessment of arteriovenous fistulas focusing on the relationships between the properties of shunted blood flow sounds and a medical/surgical history of hemodialysis patients" (Lines 5–7). Accordingly, the term "physical characteristics" used throughout the manuscript has been revised to "a medical/surgical history". (Lines 6, 80, 94-94, 111, 186-187, 210, 223, 225, 245, 287, 382)<br>• The term "events" has been revised to "AVF-related events" throughout the manuscript for clarity. (Lines 165, 188, 201, 340, 343, 344, 345,346, 354, 364, 369)<br>• We have added the explanation "Time at Maximum Power Output" at the first instance where the term "TMP" is used in the manuscript. (Line143)<br>• We have added the explanation " Maximum Power Output for each Frequency band " at the first instance where the term "MF" is used in the manuscript. (Line147)<br>We sincerely apologize for the inconvenience, but we kindly ask you to refer to the 'Revised Manuscript with Track Changes' for the other minor corrections. |
